# Increased plasma BMP-2 levels are associated with atherosclerosis burden and coronary calcification in type 2 diabetic patients

**DOI:** 10.1186/s12933-015-0214-3

**Published:** 2015-05-24

**Authors:** Ming Zhang, Jaskanwal Deep Sara, Fei-long Wang, Li-Ping Liu, Li-Xiao Su, Jing Zhe, Xi Wu, Jing-hua Liu

**Affiliations:** Department of Cardiology, Beijing Anzhen Hospital, Capital Medical University, Beijing, 100029 PR China; Beijing Institute of Heart, Lung and Blood Vessel Disease, Beijing, China; Division of Cardiovascular Diseases, Mayo College of Medicine, Rochester, MN USA; Department of Nephrology, First Hospital of Tsinghua University, Beijing, China; Department of Biostatistics, Rutgers School of Public Health, The State University of New Jersey, Piscataway, NJ USA; Department of Biostatistics, University at Buffalo, the State University of New York, Buffalo, NY 14214 USA

**Keywords:** Bone morphogenetic protein-2, Type 2 diabetes mellitus (T2DM), Coronary artery disease, Plaque burden and plaque calcification

## Abstract

**Background:**

Although Bone morphogenetic protein-2 (BMP-2) is a known mediator of bone regeneration and vascular calcification, to date no study has investigated the relationship between BMP-2 and type 2 diabetes mellitus (T2DM) and its possible role in coronary artery disease (CAD). The purpose of this study is to evaluate the relationship of BMP-2 with atherosclerosis and calcification in patients with T2DM.

**Methods:**

124 subjects were enrolled in this study: 29 patients with T2DM and CAD; 26 patients with T2DM and without CAD; 36 patients with CAD and without T2DMand 34 without T2DM or CAD (control group). Severity of coronary lesions was assessed using coronary angiography and intravascular ultrasound (IVUS). Plasma BMP-2 levels were quantified using a commercially available ELISA kit.

**Results:**

Compared to the control group, the mean plasma BMP-2 level was significantly higher in T2DM patients with or without CAD (20.1 ± 1.7 or 19.3 ± 1.5 pg/ml, vs 17.2 ± 3.3 pg/ml, P <0.001). In a multivariable linear regression analysis, both T2DM and CAD were significantly and positively associated with BMP-2 (Estimate, 0.249; standard error (SE), 0.063; *p* <0.0001; Estimate, 0.400; SE, 0.06; *p* <0.0001). Plasma BMP-2 was also strongly correlated with glycosylated hemoglobin A1c (HbA1c) (Spearman ρ = −0.31; *p* = 0.0005). SYNTAX score was also significantly associated with BMP-2 (Spearman ρ = 0.46; *p* = 0.0002). Using the results from IVUS, plasma BMP-2 levels were shown to positively correlate with plaque burden (Spearman ρ = 0.38, *P* = 0.002) and plaque calcification (Spearman ρ =0.44, P = 0.0003) and to negatively correlate with lumen volume (Spearman ρ =0.31, P = 0.01).

**Conclusions:**

Our study demonstrates that patients with T2DM had higher circulating levels of BMP-2 than normal controls. Plasma BMP-2 levels correlated positively with plaque burden and calcification in patients with T2DM.

## Introduction

Bone morphogenetic protein-2 (BMP-2), a member of the transforming growth factor (TGF) superfamily, plays a major role in the regulation of conventional and ectopic osteogenesis [1,2]. A growing body of evidence indicates that BMP-2 signaling plays an important role in vascular disease, including atherosclerosis, plaque instability [3,4], and vascular calcification and inflammation [5,6].

Specifically, increased levels of BMP-2 exert proinflammatory, proatherogenic effects by inducing oxidative stress and endothelial dysfunction and have been shown to promote plaque calcification by inducing an osteogenic phenotype in vascular smooth muscle cells (VSMCs) [7,8] In the coronary arteries, BMP-2 is regulated by inflammatory stimuli [9] and its upregulation in the vasculature may also be related to oxidative stress, hyperglycemia and hyperlipidemia [10,11].

Diabetes mellitus substantially increases the risk of cardiovascular and peripheral arterial disease [12] and is associated with an increased risk of cardiovascular mortality. People with type 2 diabetes (T2DM) have approximately a doubled risk of cardiovascular disease, compared with people without diabetes, even after adjusting for established cardiovascular risk factors [13]. The pathophysiology of vascular disease in diabetes involves abnormalities in endothelial and VSMCs, and alterations in platelet function [14,15]. Vascular calcification is also more common in patients with diabetes and is associated with increased mortality, stroke and amputations [16-19]. Highly prevalent vascular calcification in patients with T2DM may be related to biochemical changes [20,21]. For example, Chen et al. found that high serum glucose levels were associated with an increased expression of Cbfa1 and BMP-2 and enhanced the calcification of VSMCs [22,23].

Neverthesless, the role of high glucose concentrations in atherosclerosis is complex and requires clarification. In addition, while BMP-2 is a known mediator of vascular calcification [19], the role of BMP-2 in the development of atherosclerosis remains uncertain, particularly amongst T2DM patients. In this study, we measured plasma BMP-2 levels in patients with CAD and T2DM and examined the association between BMP-2 and clinicopathological parameters of coronary atherosclerotic disease.

## Methods

### Study subjects

Between 6/2/2011 and 6/17/2013, a total of 124 consecutive patients undergoing diagnostic coronary angiography for the evaluation of coronary artery disease (CAD) were recruited. The diagnosis of CAD was made using angiography and was defined as angiographic evidence of ≥50% luminal narrowing in at least one segment of a main epicardial coronary artery. Study subjects were divided into four groups: (i) patients without CAD or T2DM (n = 34); (ii) patients with T2DM only (n = 26); (iii) patients with CAD only (n =31) and (iv) patients with T2DM and CAD (n =29). Diabetes mellitus was diagnosed based upon the WHO guidelines or current intake of hypoglycemic agents [24].

Patients with a history of significant concomitant disease including hepatic failure, renal failure, hepatitis, cardiomyopathy, congenital heart disease, bleeding disorders, previous thoracic irradiation therapy, and malignant diseases were excluded from the study. This study was approved by the Beijing Anzhen Hospital Ethics Committee of Capital Medical University, and informed consent was obtained from all participants.

### Plasma collection and storage

Blood samples were collected in EDTA-containing tubes (BD, Franklin Lakes, NJ, USA) and plasma was isolated within 1 hour by centrifugation at 1900 × g for 10 minutes at 4°C to remove blood cells, and then at 16,000 × g for 10 minutes at 4°C to remove additional cellular nucleic acid attached to cell debris. Plasma samples were stored at −80°C prior to being analyzed.

### Specific BMP-2 ELISA

BMP-2 levels were quantified using a commercially available ELISA kit (Quantikine, BMP-2 Immunoassay, R&D Systems, MN USA). All samples were assayed according to the manufacturer’s instructions and were tested in duplicate by personnel blinded to each patient’s group. The optical density of each well was determined using a microplate reader at 450 nm. No interference and no cross-reactivity were expected based on the manufacturer’s instructions.

### Coronary angiography and assessment of CAD severity

Coronary angiography was performed according to standard methods. Each coronary angiogram was scored by two independent investigators according to the following scores: (1) Vessel score: The number of vessels with significant (≥50%) stenosis [25]; (2) Synergy between PCI with Taxus and Cardiac surgery score (SYNTAX score), an angiographic score that determines the severity and complexity of disease in coronary lesions of ≥50% stenosis, in vessels ≥1.5 mm [26-28]. All angiographic variables of the SYNTAX score were computed by two experienced cardiologists who were blinded to procedural and clinical data. In cases of disagreement, the final decision was reached by consensus.

### Intravascular ultrasound examination and image analysis

Virtual histology-Intravascular Ultrasound (IVUS) examinations were performed using a 20 MHz, 2.9 F phased-array IVUS catheter (Eagle Eye Gold, Volcano Corporation, Rancho Cordova, CA, USA). Vessel and lumen borders were manually contoured for all frames in each coronary segment. Quantitative IVUS measurements inclued vessel volume, lumen volume, plaque volume and percent plaque burden. Radiofrequency IVUS derived plaque components were color-coded and reported as the absolute plaque volume of VH-IVUS parameters [fibrous (dark green), fibrofatty (light green), necrotic core (red) and dense calcium (white)] to assess for plaque composition [29,30]. Simpson’s rule for volumetric measurements was used. To compensate for the different segment lengths of each analyzed artery, all volumetric data were divided by segment length and displayed as volume index (mm3/mm) [31].

### Statistical analysis

Normality of distribution was assessed using Kolmogorov-Smirnov test. Comparisons between 2 groups were performed using Fisher’s test, Student’s *t* test or Mann–Whitney *U* test. For comparisons between more than 2 groups, one-way ANOVA and Tukey-Kramer HSD (honestly significant difference) tests were used as appropriate. Pearson *χ*^2^ test and Spearman ρ test were used to compare qualitative and quantitative variables as appropriate. Correlations between BMP-2 and other variables were calculated using Pearson’s correlation coefficient for symmetrically distributed data and Spearman’s correlation coefficient for skewed variables. Univariate and multivariable linear regression analyses were performed to identify variables that were associated with BMP-2 after adjusting for potential confounders. All tests were two-sided and a *p* <0.05 was considered statistically significant. All statistical analyses were performed using JMP 10 software (SAS Institute, Inc., Cary, NC, USA).

## Results

### Baseline clinical characteristics of study participants

Among the 124 subjects, mean age was 59.4 ± 11.7 years and the proportion of males was 66%. Mean BMP-2 in the study population was 18.8 ± 2.5 (pg/ml). Of the 124 patients, 60 were diagnosed as having CAD and 45 patients had T2DM, and the remainder had neither disease (n = 34). Prevalence of hypertension, dyslipidemia and smoking was 83 (69%), 86 (71%) and 51 (42%) respectively.

## Elevated plasma BMP-2 levels are associated with the presence of T2DM

**Figure 1:**
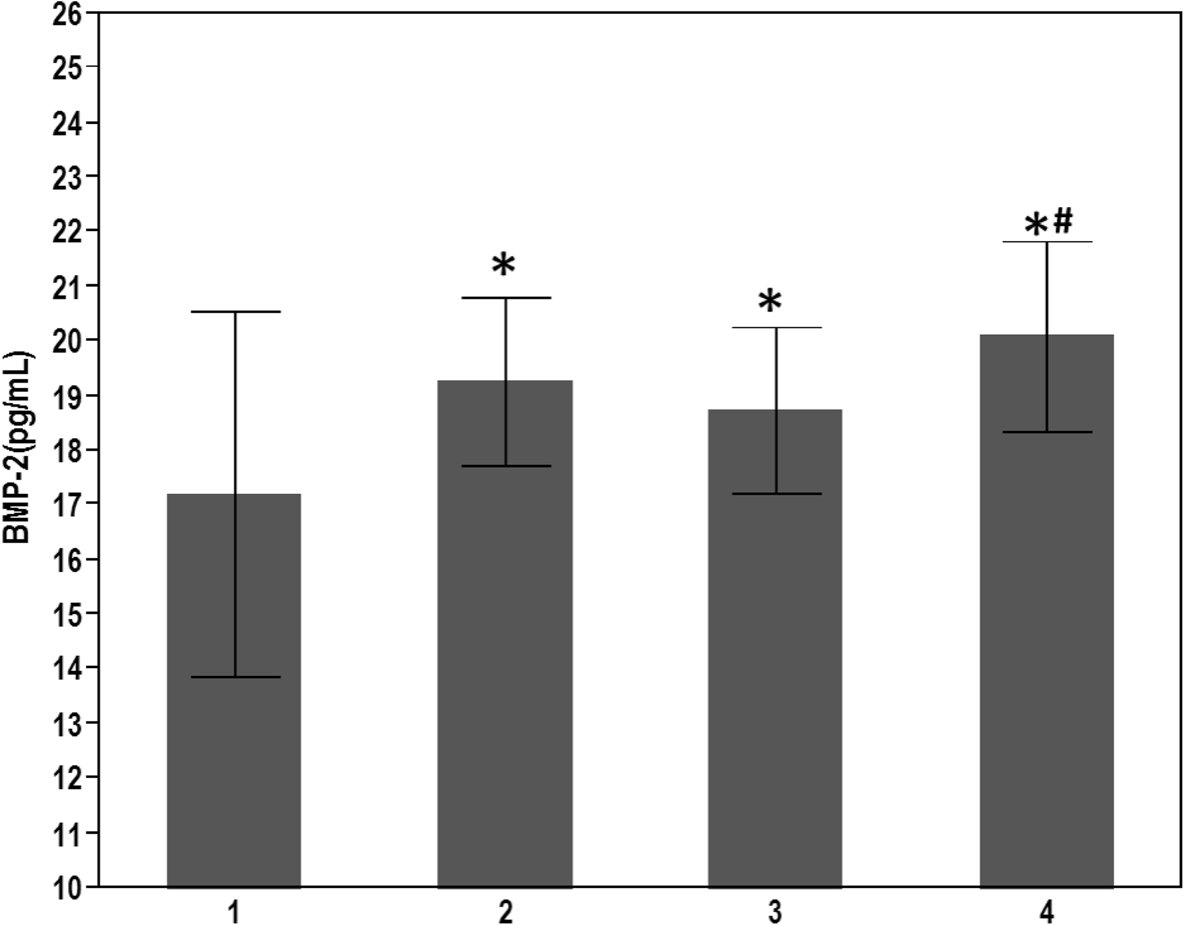
Elevated plasma BMP-2 levels are associated with the presence of DM and CAD. Bars represent average plasma BMP-2 levels amongst patients in each group. T-bars indicate standard deviation. Groups are as follows: 1; patients without CAD or T2DM, 2; patients with T2DM only; 3, patients with CAD only and 4, patients with T2DM and CAD. (*p < 0.05 as compared to group without CAD and T2DM; # p < 0.05 as compared to group with CAD only).

BMP-2 levels differed significantly across all groups (Table 1). Compared to subjects without T2DM or CAD, mean plasma BMP-2 levels were significantly higher in patients with T2DM with or without CAD (20.1 ± 1.7 or 19.3 ± 1.5 pg/ml vs 17.1 ± 3.3, P <0.001, respectively) (Table 1, Figure 1).

**Table 1:** **Baseline patient characteristics amongst all patients**

**Clinical variables**	**Normal (n = 34)**	**T2DM (n = 26)**	**CAD (n = 31)**	**T2DM + CAD (n = 29)**	**P value**
Age, yr	58.0 ± 11	60 ± 2.3	57 ± 2.2	61.6 ± 2.0	0.41
Male Gender, n (%)	21,(61)	20,(77)	21,(68)	20,(61)	0.50
Body mass index, kg/m2	26 ± 2.9	27 ± 3.7	27 ± 4.0	30 ± 3.7	0.07
Hypertension, n (%)	18(56)	21(84)	119(61)	25(76))	0.08
Dyslipidemia, n (%)	8(25)	5(21)	19(61)	25(76)	0.63
Smoking, n (%)	15(76)	12(48)	15(48)	9(27)	0.23
Blood glucose, mmol/L	5.6 ± 1.1	6.2 ± 2.2	5.5 ± 1.0	7.6 ± 2.8	0.004
Triglycerides, mmol/L	1.7 ± 1.1	2.0 ± 0.3	2.0 ± 0.2	1.9 ± 0.2	0.63
Total cholesterol, mmol/L	4.4 ± 0.2	4.3 ± 0.2	4.4 ± 0.9	4.2 ± 0.2	0.92
Low-density lipoprotein, mmol/L	2.7 ± 0.9	2.7 ± 0.8	2.7 ± 0.3	2.5 ± 0.9	0.62
High-density lipoprotein, mmol/L	1.0 ± 0.04	1.0 ± 0.04	1.0 ± 0.03	0.98 ± 0.04	0.80
Hs CRP, mg/L, Median (Q1, Q3)	1.5(0.7,3.7)	0.64(0.4,1.6)	1.2(0.7,2.9)	1.4(0.7,6.5)	<0.001
Cr, μmol/L Median (Q1, Q3)	72(65,85)	76(67,86)	76(66,88)	77(63,84)	0.9
Ejection fraction, %	64 ± 5.7	63 ± 6.7	64 ± 7.3	62.2 ± 6.2	0.8
LVEDD, mm	47 ± 4.9	50.1 ± 4.5	48 ± 5.3	47 ± 4.6	0.20
BMP-2 (pg/mL)	17.1 ± 0.6	19.2 ± 0.3	18.7 ± 0.3	20.1 ± 0.3	P < 0.001
HbA1c (n %)	5.9 ± 1.52	7.3 ± 1.6	5.8 ± 0.7	8.23 ± 1.6	P < 0.001
**Medications on admission**					
Aspirin, No. (%)	24(71)	18(69)	24(77)	26(78)	0.78
Clopidogrel, No. (%)	13(38)	12(46)	16(52)	20(61)	0.32
Beta-blockers, No. (%)	14(41)	12(48)	14(45)	16(48)	0.93
ACEI/ARB, No. (%)	14(42)	17(71)	16(76)	23(85)	0.0017
Statins, No. (%)	8(23.5)	15(58)	15(48)	22(67)	0.0025
Insulin, No. (%)	0(0)	17(65)	0(0)	23(79)	<0.001
Oral hypoglycemics, No. (%)	0(0)	18(69)	0(0)	25(86)	<0.001

BMP-2, Bone Morphogenic Protein-2; CAD, Coronary artery disease; HbA1c; Glycosylated hemoglobin A1c; hsCRP, High sensitivity C-reactive protein; LVEDD, Left ventricular end diastolic diameter; Cr, Serum creatinine; T2DM, Type II Diabetes Mellitus.

**Figure 2:**
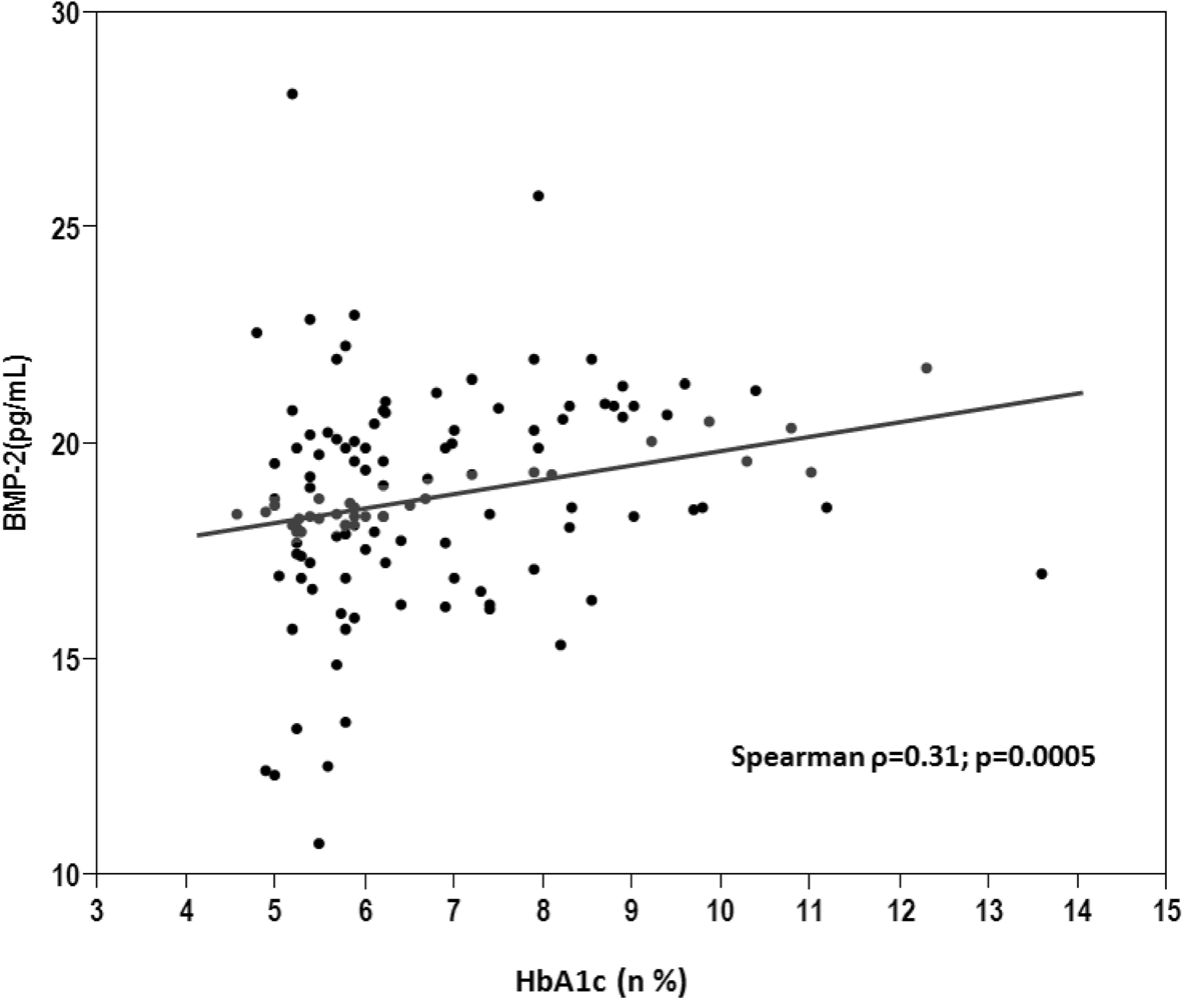
Scatter diagram demonstrating the correlation between BMP-2 levels and HbA1c. HbA1c is significantly positively correlated with plasma BMP-2 levels.

Table 2 demonstrates the results of an univariate linear regression analyses to determine the association between clinical variables and BMP-2. Random blood glucose levels, and the presence of T2DM and CAD were significantly associated with BMP-2. High-sensitivity C-reactive protein showed a tendency towards a positive association with BMP-2 with borderline statistical significance. After adjustment for traditional cardiovascular risk factors, random blood glucose, HbA1c, T2DM and CAD were significantly associated with BMP-2 levels (Estimate, 0.32; standard error, 0.16; *p* =0.04; Estimate, 0.30; standard error, 0.13; *p* =0.021; Estimate, 1.565; standard error, 0.434; *p* =0.0005; Estimate, 1.299; standard error, 0.425; *p* =0.0028, respectively) (Table 3). An interaction term between CAD and T2DM was included in our multivariable analysis and proved not to be significant (p = 0.46) and so was excluded from our final model. Plasma BMP-2 levels correlated positively with HbA1c (Spearman ρ = 0.31; *p* = 0.0005) (Figure 2).

**Table 2:** **Association between BMP-2 with type II diabetes mellitus and coronary artery disease after adjusting for traditional cardiovascular risk factors**

**Variable**	**Estimate**	**Standard error**	**P**
Age (Increase by 1 year)	0.57	0.42	0.19
Male gender	−0.032	0.07	0.68
Body mass index	−0.01	0.009	0.30
Hypertension	−0.11	0.08	0.18
Dyslipidemia	−0.006	0.08	0.94
Smoking	−0.051	0.08	0.50
Random blood glucose, mmol/L (Increase by 1 mmol/L)	0.12	0.05	0.027
Hs-CRP, mg/L (Increase by 1 mg/L)	0.33	0.19	0.08
HbA1c (n %) (Increase by 1%)	0.17	0.06	0.0079
T2DM	−0.39	0.11	0.0002
CAD	−0.26	0.09	0.004

Data are expressed as parameter estimates with standard errors. CAD, Coronary artery disease; HbA1C, glycosylated hemoglobin A1C; Hs-CRP, Highly sensitive C-reactive protein; T2DM, Type II diabetes mellitus.

**Table 3:** **Association between BMP-2 levels and plaque volume and dense calcium in patients with coronary artery disease**

**Variable**	**Adjusted for traditional risk**		
	**Estimate**	**SE**	**P**
T2DM	1.565	0.434	0.0005
CAD	1.299	0.425	0.0028
Random blood glucose, mmol/l (Increase by 1 mmol/l)	0.32	0.160	0.04
HbA1c, %	0.30	0.13	0.021

CAD, Coronary artery disease; HbA1C, glycosylated hemoglobin; T2DM, Type II diabetes mellitus.

### Assessment of CAD severity using coronary angiography

**Figure 3:**
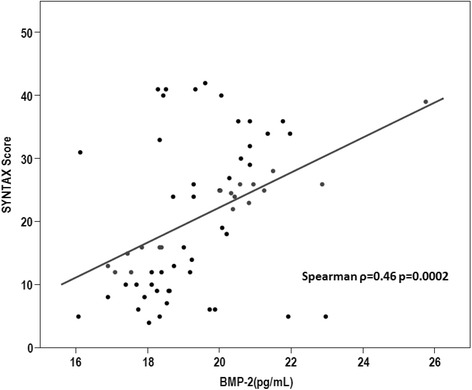
Scatter diagram demonstrating the correlation between BMP-2 levels and SYNTAX score. SYNTAX is significantly positively correlated with plasma BMP-2 levels.

CAD with three-vessel disease was more prevalent in the CAD and T2DM group compared with the CAD only group (48.9% vs 20.6%, *p* = 0.002). When assessing the severity of CAD using the SYNTAX score, the mean SYNTAX score in CAD patients with T2DM were significantly higher than in patients with CAD only (29.3 + 6.3 vs. 11.7 + 6.2, *p* < 0.001). Plasma levels of BMP-2 correlated positively with SYNTAX score (Spearman ρ = 0.46; *p* = 0.0002) (Figure 3). In a multivariable linear regression analysis, SYNTAX score was significantly associated with BMP-2 levels (Estimate, 2.5, standard error, 0.79; *p* = 0.0026) (Table 4).

**Table 4:** **Virtual Histology-Intravascular Ultrasound parameters amongst study groups**

**Variable**	**Univariate**			**Adjusted for risk factors**		
	**Estimate**	**SE**	**P**	**Estimate**	**SE**	**P**
SYNTAX score	2.78	0.76	0.0005	2.5	0.79	0.0026
Lumen volume index, (mm3/mm)	−0.34	0.13	0.0126	−0.273	0.13	0.042
Plaque volume index, (mm3/mm)	0.61	0.25	0.018	0.58	0.27	0.035
Dense calcium, (mm3/mm)	0.29	0.09	0.0028	0.31	0.10	0.0025

CAD, Coronary artery disease; SE, Standard error; Data are expressed as parameter estimates with standard errors. Risk factors adjusted for in multivariate analysis: age, gender, hypertension, hyperlipidemia and smoking.

### Assessment of plaque burden and calcium density using intravascular ultrasound

Data derived from IVUS are shown in Table 5. Plaque volume index and dense calcium were significantly higher in patients with CAD and T2DM compared to patients with CAD only (13.9 [12,15] vs. 9.3 [8.2, 12]; *p* < 0.0001; 2.7 [2.1, 3.8] vs. 2.0 [1.3, 3.0]; *p* = 0.023). Lumen volume index in the CAD and T2DM group was significantly lower compared with that in the CAD group (4.4 [3.8, 6.5] vs. 5.8 [4.8, 6.5]; *p* < 0.001) (Table 5).

**Table 5:** **Univariate linear regression analysis for plasma levels of BMP-2**

**IVUS parameter**	**CAD (n = 31)**	**T2DM + CAD (n = 29)**	**P value**
IVUS target vessel			0.51
LAD	17(55)	17(51)	
RCA	9(29)	7(21)	
LCX	5(16)	9(27)	
IVUS vessel length(mm) Median (Q1, Q3)	24 (17, 35)	21 (14, 28)	0.22
Lumen volume index, (mm3/mm) Median (Q1, Q3)	5.8 (4.8, 7.4)	4.4 (3.8, 6.5)	0.0051
Plaque volume index, (mm3/mm) Median (Q1, Q3)	9.3 (8.2, 12)	13.9 (12, 15)	<0.001
Dense calcium, (mm3/mm), Median (Q1, Q3)	2 (1.3, 3)	2.7 (2.1, 3.8)	0.023
Fibrotic tissue, (mm3/mm), Median (Q1, Q3)	2.8 (1.8, 4)	3.9 (2.7, 5.9)	0.82
Necrotic core, (mm3/mm), Median (Q1, Q3)	2.3 (1.5, 3.5)	2.8 (2.2, 4)	0.06
Fibro-fatty, (mm3/mm) , Median (Q1, Q3)	0.5 (0.1, 0.7)	0.3 (0.1, 0.75)	0.91

In a multivariable linear regression analysis, lumen volume, plaque volume index and plaque dense calcium was significantly associated with BMP-2 (Estimate, −0.273; standard error, 0.13; *p* = 0.042 and Estimate, 0.58; standard error, 0.27; *p* = 0.035; Estimate, 0.31; standard error, 0.10; *p* = 0.0025, respectively) (Table 4).

**Figure 4:**
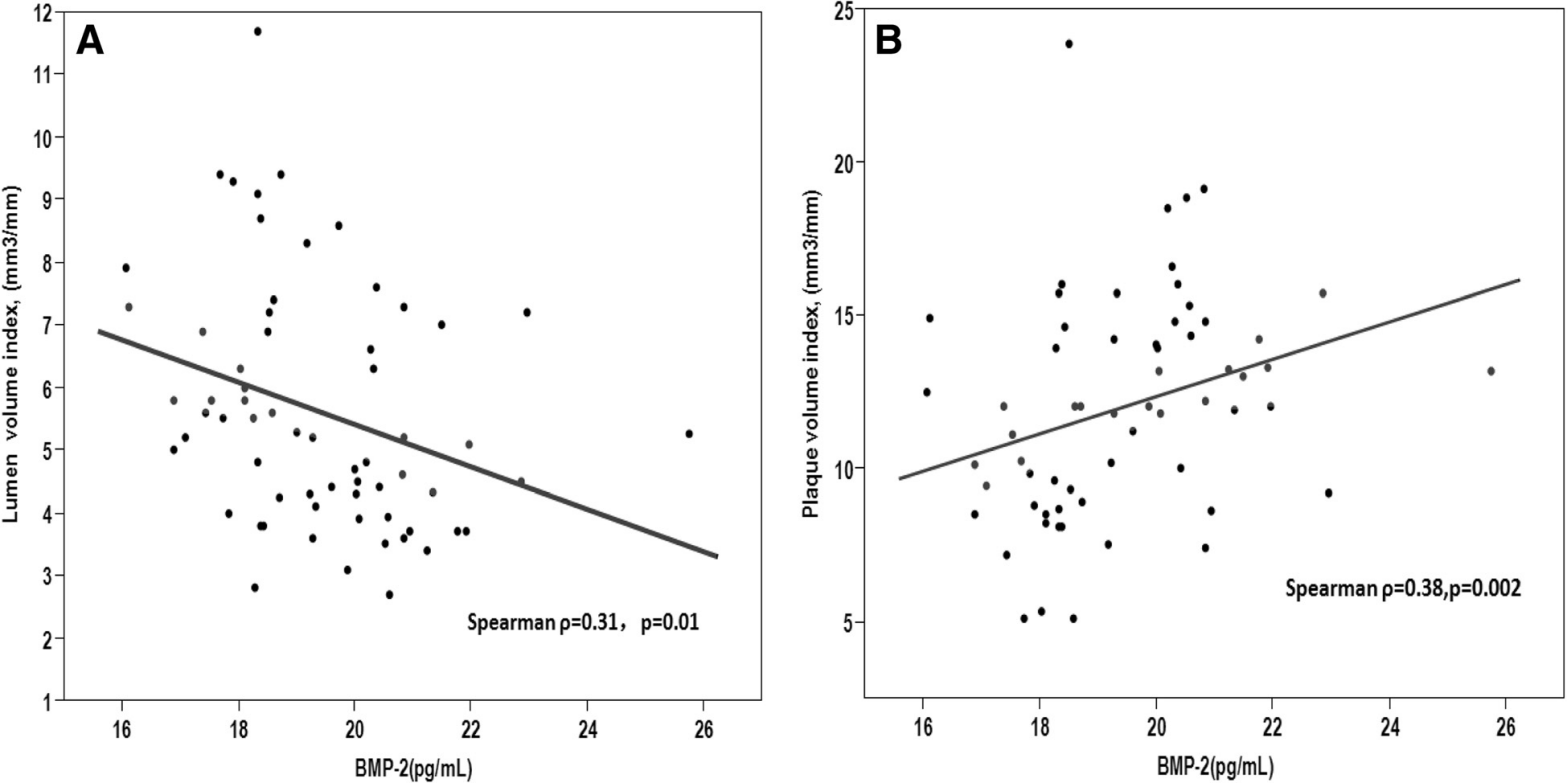
Scatter diagram demonstrating the correlation between BMP-2 levels and lumen volume index, and plaque volume index. **(A)** Plasma BMP-2 is significantly negatively correlated with lumen volume index. **(B)** Plasma BMP-2 is significantly positively correlated with plaque volume index.

**Figure 5:**
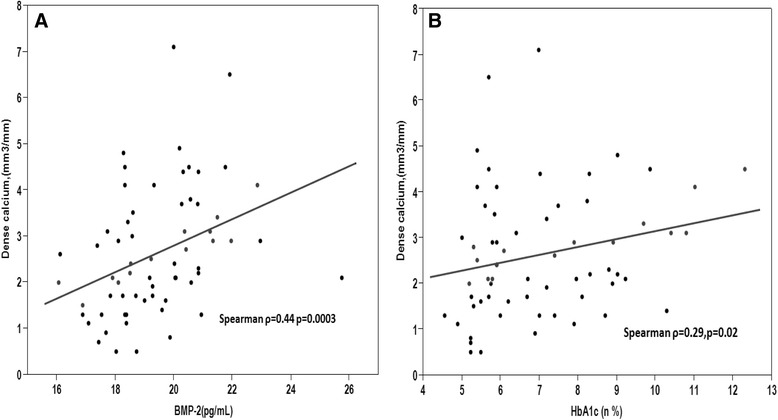
Scatter diagram demonstrating the correlation between BMP-2 levels and plaque dense calcium, and the association between HbA1c and plaque dense calcium. **(A)** Plasma BMP-2 is significantly positively correlated with plaque dense calcium. **(B)** HbA1c is significantly positively correlated with plaque dense calcium.

Plasma BMP-2 levels correlated negatively with lumen volume index (Spearman ρ = −0.31, *P* =0.01) (Figure 4A), and correlated positively with plaque volume index (Figure 4B), and plaque calcium density (Spearman ρ =0.38, *P* =0.002; Spearman ρ =0.44, *P* =0.0003, respectively) (Figure 5A). Dense calcium correlated positively with plasma HbA1c levels. (Spearman ρ =0.29, *p* = 0.02) (Figure 5B).

## Discussion

In the current study, we examined the association between plasma BMP-2 levels and CAD in patients with and without T2DM. Compared to normal subjects, plasma BMP-2 levels were significantly higher in T2DM patients with and without CAD. Plasma BMP-2 levels also correlated positively with random blood glucose levels and HbA1c. In addition, plasma BMP-2 levels were significantly higher in patients with CAD compared to those without CAD and were positively associated with the severity and extent of coronary lesions. Our IVUS findings also showed that plasma BMP-2 levels correlated positively with plaque calcification and plaque burden, which suggests an important role of BMP-2 in accelerating atherosclerotic progression and plaque calcification.

### BMP-2 is associated with CAD in patients with T2DM

Recent studies have demonstrated that BMP-2 plays an important role in both physiological and pathological vascular processes [32,33]. VSMCs represent a significant source of BMP-2, which has been reported to be highly expressed in human vessels [34,35]. Moreover, several studies have consistently shown that BMP-2 enhances VSMC migration [36,37]. Hyperglycemia plays a key role in the regulation of the vascular inflammatory response by activating the recruitment of inflammatory cells to injured arteries. Hyperglycemia also stimulates atherosclerosis through various processes including endothelial dysfunction and cellular proliferation as well as inflammation [22,38,39]. Although the role of high serum glucose levels in atherosclerosis is well documented, its role in vascular calcification is more complex. Our previous study showed that high glucose levels increased BMP-2 expression, concomitantly with NF-kB activation [23]. In the current study, we extend these findings by showing that plasma BMP-2 levels correlate positively with HbA1c, suggesting that chronically elevated glucose levels may enhance BMP-2 expression in patients with T2DM.

### The correlation between BMP-2 and coronary artery disease

Although BMP-2 is important for bone regeneration and is a known mediator of vascular calcification, its role in the regulation of atherosclerotic lesions remains uncertain. BMP-2 exerts proinflammatory, proatherogenic effects and can cause oxidative stress in endothelial cells promoting endothelial cell activation [37]. The current study demonstrates that plasma levels of BMP-2 are significantly higher in patients with CAD. In addition, we showed that BMP-2 levels are positively associated with the severity and extent of coronary stenotic lesions as measured using the SYNTAX score. These results suggest that BMP-2 may play a key role in the pathogenesis of coronary atherosclerosis.

### BMP-2 may be involved in hyperglycemia-induced plaque calcification

Vascular calcification is commonly found in atherosclerosis and is recognized as a marker of plaque burden. The presence of spotty calcification is associated with more extensive and diffuse coronary atherosclerosis and accelerated disease progression despite the use of medical therapy [40]. Arterial stiffness has been shown to be an independent predictor of cardiovascular mortality and an early marker of target organ damage by cardiovascular disease. Vascular calcification is as a major contributor to loss of arterial compliance [41].

Vascular calcification is common in diabetes and is associated with high risk of cardiovascular events. Lesion length, plaque burden, necrotic core, and calcium content have all been shown to be significantly higher in nonculprit lesions of diabetic patients [42]. Though it is known that diabetes is associated with increased vascular calcification, the pathogenesis is not completely understood. VSMCs are thought to drive the calcification process [43]. Elevated glucose levels have been shown to direct osteogenesis by transforming VSMCs and possibly pericytes into osteoblast-like cells [44]. In vitro studies have also shown that high-glucose concentrations are associated with the increased expression of Cbfa1 and osteocalcin as well as the increased activity of alkaline phosphatase VSMCs. These changes resulted in enhanced calcification and cell proliferation and the increased expression of osteopontin in cultured VSMCs [22,45] as well as in the medial layers of the carotid arteries of streptozotocin-induced diabetic rats [46]. Bostrom and Chen et al. also demonstrated that BMP-2 increased calcification and osteogenic differentiation in calcifying VSMCs, and that exogenous BMP-2 significantly increased the calcification in VSMCs [21,47]. In this study, our IVUS finding showed that plasma BMP-2 levels correlated positively with plaque dense calcium, which itself correlated positively with plasma HbA1c. These findings suggests that BMP-2 may have a role in hyperglycemia-induced calcification, though the precise molecular mechanisms involved require further study.

### Limitations

This study was limited by a small sample size that presented to a University Hospital, likely to constitute a unique cohort, which may restrict the generalizability of our results. The cross-sectional design of the study also makes determining a causal relationship between BMP-2 and CAD challenging. Further prospective studies are required to determine the role that elevated BMP-2 levels have on clinical outcomes in patients with CAD.

## Conclusion

BMP-2 levels are increased in CAD patients with T2DM and correlate positively with the extent and complexity of coronary atherosclerotic disease as well as the degree of plaque calcification. These finding suggest that BMP-2 may be an important mediator of hyperglycemia-induced plaque progression and calcification.
